# Does the process of developing products for knowledge mobilisation from healthcare research influence their uptake? A comparative case study

**DOI:** 10.1186/s43058-022-00360-9

**Published:** 2022-12-14

**Authors:** Charlotte A. Sharp, Ruth J. Boaden, William G. Dixon, Caroline Sanders

**Affiliations:** 1 The Centre for Epidemiology Versus Arthritis, The University of Manchester, Stopford Building, Oxford Road, Manchester, M13 9PG UK; 2 The Centre for Primary Care and Health Services Research + NIHR Greater Manchester Patient Safety Translational Research Centre, The University of Manchester, The Williamson Building, Manchester, M13 9PT UK; 3 Salford Royal NHS Foundation Trust, Northern Care Alliance, Salford, UK; 4 Alliance Manchester Business School, Booth Street West, Manchester, M15 6PB UK

**Keywords:** Knowledge mobilisation, Applied healthcare research, Implementation, Qualitative, Products, Dissemination, Toolkits

## Abstract

**Background:**

Getting knowledge from healthcare research into practice (knowledge mobilisation) remains a global challenge. One way in which researchers may attempt to do this is to develop products (such as toolkits, actionable tools, dashboards, guidance, audit tools, protocols and clinical decision aids) in addition to journal papers. Despite their increasing ubiquity, the development of such products remains under-explored in the academic literature. This study aimed to further this understanding by exploring the development of products from healthcare research and how the process of their development might influence their potential application.

**Methods:**

This study compared the data generated from a prospective, longitudinal, comparative case study of four research projects which aimed to develop products from healthcare research. Qualitative methods included thematic analysis of data generated from semi-structured interviews (38), meeting observations (83 h) and project documents (300+). Cases were studied for an average of 11.5 months (range 8–19 months).

**Results:**

Case comparison resulted in the identification of three main themes with the potential to affect the use of products in practice. First, *aspects of the product*, including the perceived need for the specific product being identified, the clarity of product aim and clarity and range of end-users. Second, *aspects of development*, whereby different types of stakeholder engagement appear to influence potential product application, which either needs to be ‘meaningful’, or delivered through the implicit understanding of users’ needs by the developing team. The third, overarching theme, relates to the *academic context* in which products are developed, highlighting how the academic context perpetuates the development of products, which may not always be useful in practice.

**Conclusions:**

This study showed that aspects of products from healthcare research (need/aim/end-user) and aspects of their development (stakeholder engagement/implicit understanding of end-users) influence their potential application. It explored the motivation for product development and identifies the influence of the current academic context on product development. It shows that there is a tension between ideal ‘systems approaches’ to knowledge mobilisation and ‘linear approaches’, which appear to be more pervasive in practice currently. The development of fewer, high-quality products which fulfil the needs of specified end-users might act to counter the current cynicism felt by many stakeholders in regard to products from healthcare research.

**Supplementary Information:**

The online version contains supplementary material available at 10.1186/s43058-022-00360-9.

Contributions to the literature

- This is a novel comparative case study which explores the development of products from healthcare research and is important because the development of products is likely to continue to increase, with associated costs for research funders, researchers and stakeholders.
- The tension between the development of such products as a first generation (linear) solution to a problem perhaps better addressed by third generation (systems) approaches is highlighted.
- Having a perceived need for the product, clear aims and intended users and paying attention to stakeholder engagement influence the potential of products to mobilise knowledge from healthcare research

## Background

The rise in evidence-based practice in healthcare [1] has been accompanied by challenges not only in developing the best possible evidence, but also in ensuring that evidence is acted upon appropriately and in a timely fashion. Researchers have long been required to develop journal articles and conference proceedings in order to fulfil expectations set by higher education institutions and funders. However, such academic papers and conference proceedings have limited use in changing practice, and so attention has turned in recent years to demonstrating the ‘impact’ of research findings upon practice [2]. Researchers are now obliged to demonstrate that they have considered dissemination of research findings ‘beyond publication’ [3], with funders expecting them to show how dissemination will contribute to getting the findings into practice [4, 5]. How ‘research-based knowledge is accessed, applied and embedded’ [6] into practice using collaboration and engagement with stakeholders throughout the research process and beyond may be referred to as ‘knowledge mobilisation’. This paper focuses on the mobilisation of research findings into practice through the development, and use, of research products.

### Products from healthcare research

Researchers have responded to the challenge of mobilising knowledge from healthcare research, in part, by developing ‘products’, which may encourage users to perform specific action(s) and therefore enable research findings to be used more widely in practice [7]. Examples of research products include toolkits [8], actionable tools [9], dashboards [10], guidance, audit tools, protocols and clinical decision aids [11].

There has been a steady rise in the development of research products in recent years. The continuation of this upwards trend appears inevitable given that funders ask increasingly for their inclusion in research design [12], with some calling for this practice to persist [11]. A large multi-stakeholder interview study found that ‘creating and sharing products had an attraction and a momentum that was irresistible’ [13]. Despite this growth, there is a paucity of evidence surrounding the development and use of research products, although quality improvement toolkits have been explored to some extent [14, 15].

**Fig. 1:**
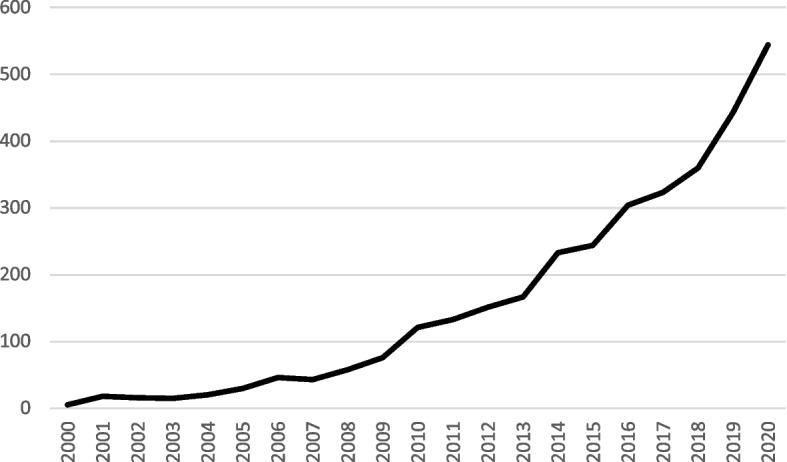
Rise in citations on PubMed including the terms ‘toolkit*’ AND ‘health*’ 2000–2020 [accessed 10/11/2021]

One example of products which aim to mobilise knowledge from healthcare research is toolkits. Using toolkits as an example of research products helps to illustrate a research gap in our understanding of product development and subsequent application, and it is reasonable to assume that similar findings would apply to other products. The development of toolkits in healthcare is rising, exemplified by increasing citations including the terms ‘toolkit*’ and ‘health*’ in major health research databases such as PubMed over the last 20 years (Fig. 1).

Evidence is lacking regarding both the development and use of research products such as toolkits. Previous qualitative studies have focussed solely on the views of end-users [12], with others simply including a description of product development by their developers (e.g. Goyder [16]). A scoping review of 83 ‘health-based’ toolkits found that information regarding their development is very scarce [17]. Davis and colleagues’ qualitative research study [12] contributes useful learning to the scant commentary regarding end-users’ perceptions of obesity management intervention toolkits, identifying ‘a paucity of research on the application of toolkits’ (p. 504). Their study focussed on primary and community care clinicians’ and administrators’ opinions of features and use of these products, rather than considering why or how they might best be developed. A systematic review of the effectiveness of toolkits as ‘knowledge translation strategies’ included studies which (1) evaluated toolkit effectiveness in supporting integration of evidence into practice, (2) aimed to inform or change practice, and (3) which included a comparison group. Of the 39 studies included, eight were rated as methodologically moderate-strong, with clinical outcomes ‘that could be somewhat attributed to the toolkit’ ([8], p.1). The authors concluded that more systematic approaches to developing and evaluating toolkits were required. Some scholars have called for further research to ensure that the ongoing development of toolkits is ‘both effective and a defensible investment of time and resources’ ([17], p.128).

An exploratory study, which informed the design of the research reported in this paper, used qualitative methods to explore the perspectives of a range of stakeholders regarding the development of toolkits which were designed to mobilise knowledge from healthcare research [18]. Participants reported that toolkits were more likely to be used in practice if they meet a pre-established need and are developed in collaboration with stakeholders and as part of a broader strategy of knowledge mobilisation [18]. When designing the case comparison study reported here, the decision to expand out from studying ‘toolkits’ (as in the exploratory study) to ‘products’ was informed by the difficulty in defining toolkits clearly [8, 17, 18] and a recognition that a range of products appeared to fulfil the same function.

To summarise, little evidence is available regarding why products from healthcare research are developed, how they are developed or how their development might influence their use. This area is of importance because of the potential for research waste [19, 20] if research findings are not mobilised into practice and/or research products which are developed for this purpose fail to achieve their aim.

### Models of knowledge mobilisation

The concept of knowledge mobilisation arose from the field known as dissemination, defined as the tailoring and targeting of messages arising from research to its intended audience [21]. Proponents of knowledge mobilisation emphasise the importance of long-term collaborations between researchers and research users, the influence of users upon research questions and a reciprocally enhanced understanding of research by end-users and end-users by researchers, in turn [13, 22]. Best and Holmes [23] conceptualise different perspectives on getting knowledge into action as (1) linear models, (2) relationship models and (3) systems models. This paper uses their ‘three-generations’ perspective to provide theoretical insight into the process of trying to mobilise knowledge from healthcare research (to which they refer as ‘knowledge to action’).

First generation, linear models, include ‘producer-push’ (e.g. traditional academic dissemination through academic papers) and ‘user-pull’ (e.g. knowledge ‘users’ commissioning a systematic review) [24]. Second generation, or relationship models, incorporate and build upon linear models, focussing on interactions occurring amongst knowledge users and creators [25–28], for example, linkage and exchange, and paying attention to the knowledge that arises from outside of research [29]. Third generation, systems models, encompass and builds upon linear and relational approaches. Systems models view knowledge as situated and dependent on context [25, 30, 31]. The role of collaboration with stakeholders invested in that knowledge is held in high esteem [32]. Best and Holmes’ theoretical approach is summarised in Table 1.

**Table 1:** Best and Holmes’ (2010) [23] theoretical approach to knowledge mobilisation

**Generation**	First	Second	Third
**Model**	**Linear**	**Relational**	**Systems**
**Description**	Emphasis on researcher passing knowledge on to the user	Emphasis on interactions between knowledge creators and users, with attention paid to knowledge arising outside research	Emphasis on knowledge as situated, dependent on context, and interactions with stakeholders
**Example**	Traditional dissemination activities, e.g. academic papers	Linkage and exchange, e.g. engaging opinion leaders	Active collaboration with stakeholders across the system, e.g. stakeholder engagement throughout the research cycle

This study aimed to fill the research gap identified in the literature, seeking to understand *why and how products from healthcare research are developed, and whether aspects of the product or their development might influence their potential application*. It used a prospective, longitudinal, comparative case study design to explore the development and potential use of products from healthcare research. Evaluation of the potential use of each product was based upon objective measurements (website hits) and stakeholder opinion of current and future utilisation [33]. By using Best and Holmes’s [23] systems thinking model of knowledge mobilisation, a theoretically informed approach to the analysis was taken [34].

## Methods

This study drew upon qualitative data generated from a prospective, longitudinal, comparative case study of research projects aiming to develop products from healthcare research (four cases from a large UK Russell Group University). Its reporting follows the COREQ guidelines [35] (Additional file 1).

### Study design

This prospective, longitudinal, comparative case study aimed to understand why and how products are developed, and which aspects of the products or their development might influence their potential application. The comparative case study approach enabled the in-depth study of complex contemporaneous phenomena [36, 37] and the development of broader generalisations [38–40]. This approach has been advocated for the study of dissemination and implementation research [31, 41] more broadly and knowledge mobilisation more specifically [23, 42].

Each case study followed the development of ‘products’ which aimed to help mobilise research knowledge. When designing the study described in this paper, a number of applied healthcare research projects based at the study site planned to develop products for knowledge mobilisation. Four cases were identified using a purposive and pragmatic approach [38, 43], selected for their attributes, typicality and feasibility in being able to answer the research question and provide sufficient data from which to draw meaningful comparisons [44]. Of six possible cases, one was not awarded funding in time for this research, and it emerged that another was not planning to develop an output that might be regarded as a product from healthcare research, despite initial impressions. All four cases that were approached agreed to participate.

A combination of qualitative methods was employed including semi-structured interviews with stakeholders involved in each of the projects, within-project observations and document analysis. Principal investigators (PIs) were interviewed longitudinally (0, 6, 12 months). Project managers, researchers, stakeholders, funders and organisational leads were sampled purposively, aiming to capture a range of perspectives on the same process, and interviewed once.

The University of Manchester’s Research Ethics Committee approved the study, reference 2017-2556-3819. Health Research Authority approval was awarded to cover interactions with National Health Service (NHS) employees (Integrated Research Application System reference 235035). Informed consent was written (interviews) or implied (observations).

### Data generation

Interview participants were approached via email. Participants chose interview timing and location (majority face-to-face, remainder by telephone/video conference). Interactions were recorded digitally and transcribed verbatim. Contemporaneous reflexive notes were made. Case study topic guides (Appendix 1) were informed by exploratory study findings and modified iteratively [45]. All interview participants were invited to review transcriptions, redact data and review direct quotes. Three participants chose to review the full transcripts, with two redacting a small amount of information (relating to personal relationships and not affecting the findings). Formal observations of key project meetings, stakeholder interactions and events showcasing products were participant/non-participant [46] according to each case’s preference, with notes taken contemporaneously using a proforma.

### Data analysis

The final coding framework from the exploratory study was entered into NVivo a priori (Appendix 2). An abductive approach to the thematic analysis used ‘deduction and induction to produce theoretical and empirical insights’ ([47], p. 320), building upon themes generated from the exploratory study, and the literature. Transcripts were checked contemporaneously alongside the completion of ‘contact summary forms’ ([45], p.54) to identify and reflect upon initial themes. Due to the time burden of studying four cases simultaneously, data analysis occurred following the completion of most interviews.

Because an approach acknowledging human interactions and allowing theoretical generation was required to answer the research question, a ‘weak’ social constructivist stance (which views reality as constructed as a result of humans interacting with each other and their contexts [48]), was taken. Triangulation of data across the sources looked for alignment or tension between what was observed in meetings and said during interviews and documented [47, 49]. The longitudinal interviews aided comparison between hopes, expectations and delivery of each product. A ‘summary label’ for each case regarding early use of the products was generated to aid case comparison, in an approach taken previously [33]. The analysis was discussed at three-weekly peer-debriefing sessions [50], with final themes and summary labels agreed upon by the consensus team, which comprised all four authors.

## Results

### Participants

**Fig. 2:**
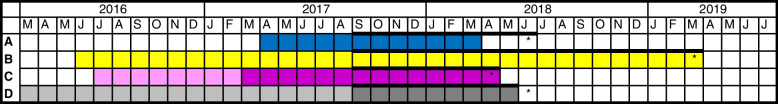
Timeline of observations for each case (Where the development ± evaluation of the product were a component of the overall project, they are shown in darker colour. The formal observation period is represented by a black line. NB: for case C, engagement with the project began in June 2016 as the main researcher was part of the team prior to studying this case). *Launch of product (individual text mining tool for case C)

All four cases were based at The University and already underway in some form when the observations began in September 2017 (Fig. 2). Cases were studied for an average of 11.5 months (range 8–19 months).

Data were generated from 38 semi-structured interviews with 30 participants, 83 h of observation and analysis of ~ 300 documents (Table 2). Of the 41 individuals approached for an interview, three declined. Interviews lasted a mean of 51 min (range 27–82) and were conducted from November 2017 to March 2019. PIs, researchers (R) and project managers (PM) were based at the university; most stakeholders (S), funders (F) and senior leaders (L) were based at large external organisations, providing a national perspective. Three respondents were familiar with two cases (either because of stakeholder engagement in both cases or due to awareness of the work being done in this field), allowing comparison. No one objected to meeting observations. Documents were sourced from research project files and emails shared with the lead researcher.

**Table 2:** Data sources

Interaction	Case				Total
	A	B	C	D	
**Interview** *Participants (interviews)*	9 (11)	11 (13)	7 (9)	6 (8)	**30**^**a**^**(38)**^**a**^
**Observations** *Number (h)*	13 (25.5)	47 (37.5)	5 (6.5)	14 (13.5)	**79 (83)**
**Documents** *Number*	44	64	102	79	**289**

^a^Three interviewees were able to comment on data across two cases, hence the final number of participants not being the sum of the figures shown

**Fig. 3:**
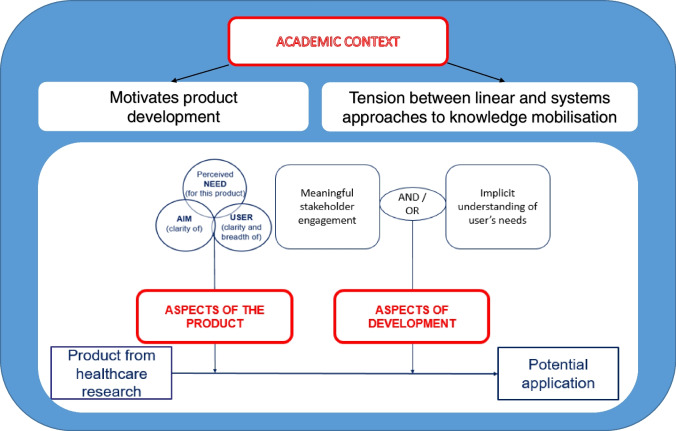
Main themes influencing the development of products from healthcare research

Three main themes were identified with the potential to affect the use of products in practice. First, *aspects of the product*, including the perceived need for the specific product being identified, the clarity of product aim, and clarity and range of end-users. These aspects were interdependent, with no fixed order required. Second, regarding *aspects of development*, different types of stakeholder engagement appear to influence potential application, which either needed to be ‘meaningful’ or delivered through the implicit understanding of users’ needs by the developing team. A third, overarching theme relates to the *academic context* in which products are developed, which includes the motivations for product development, and acknowledges the tension arising from the academic context between linear and systems approaches to knowledge mobilisation. Figure 3 summarises the overarching themes.

### Case narratives

The case narratives explore the main themes and explain the summary label framing each case. Detailed descriptions of each case and a summary of the themes are presented in Table 3. The key for the quotes is Case_Role_Interview number (PI only), i.e. A_PI_1^st^.

**Table 3:** Case comparison summary

Characteristic	Case			
	A	B	C	D
**Academic context**				
**Product aim**	To aid clinicians caring for patients with syndrome A	To enable primary care practitioners to compare local with national prescribing habits regarding drug B	To support the collection, processing and presentation of patient experience data	To encourage the use of evidence
**Project funder**	Collaboration of AHSNs, regional applied health research organisation and national charitable campaign	Regional grant funded by the government	National funding agency	National funding charity
**Product host**	National professional organisation	The university	NHS sites Text mining tool hosted by the university	The university
**Product delivered**	Yes	Product delivered but with less functionality than planned	Bespoke tools for each NHS site No cohesive, publicly available product	Yes
**Aspects of the product**				
**Perceived need**	Needed	Something is needed—is this it?	Unclear	Unclear
**Clarity of product aim**	Well defined	Well defined	Complex	Unclear
**Type of product aim**	Instrumental	Instrumental	More conceptual	Definitely conceptual
**Intended user**	Primary care practitioners	Primary care practitioners	Patient experience teams	Evaluators and decision-makers
**Clarity of user**	Clear	Clear	Clear	Contested
**Range of user group**	Narrow	Narrow	Diffuse	Broad
**Aspects of development**				
**Vision for stakeholder engagement**	Project team to consist of key stakeholders Planned to formulate product based on learning event and to obtain user feedback	Preliminary engagement planned clearly Subsequent engagement lacked strategy	Clear vision embedded throughout the project Co-design of bespoke tools for each site through qualitative interviews, focus groups and observation of tools in practice, with subsequent formal evaluation	Clear plans for interviews and a focus group to elicit feedback
**Delivery of stakeholder engagement**	Project team all contributed to the product development Little stakeholder engagement outside the core project team No changes made after learning event No user feedback	Initial stakeholder event contributed to the original vision for the product Influential stakeholders recruited to shape and champion product Regular interactions with stakeholders were not maintained and feedback not systematically assimilated/integrated into the product	Delivered	Reduced number of interactions (fewer interviews, no focus group) End of study workshop Iterative changes to the product based on interviews and end-of-study workshop
**Summary of stakeholder engagement (see Table **4** for the expansion of terms)**	Embedded	Tokenistic	Co-design	Consultation
**Role of the main researcher**	Participant observer	Observer	Participant observer	Observer, consulted formally
**Product application**				
**Perception of use in practice**	Well regarded	Regarded as potentially useful but lacking key attributes desired by users	Variable use across NHS sites participating in the project	Regarded as interesting but unlikely to be used in practice
**Web hits (12 months)**	3268	Not yet available	No data available on hits for the text mining tool	151 (downloads) 202 (online)
**Trajectory of website hits (12 months)**	Stable	N/A	N/A	Reducing
**Summary label regarding use**	**‘Is being used’**	**‘Might be used’**	**‘Some tools used locally’**	**‘Appears not to be used’**

*AHSN* Academic Health Science Network

#### Case A

Case A developed a product aiming to aid clinicians caring for patients with syndrome A, as the sole output of a 12-month project. A clinical academic PI recruited several organisations and associated personnel, who had implicit knowledge of the subject and requirements of end-users, to collaborate on product development and contribute to its funding. The PI was motivated to develop the product because syndrome A was a national clinical priority with an evident need for educational resources. The product was developed in collaboration with, and hosted by, a national not-for-profit professional organisation. At the point of the original application, the PI thought that the host organisation would fund it, akin to grant funding. However, in practice, the host organisation charged for product development and dissemination.

Aside from early work which helped form the key themes for the product, very little stakeholder engagement occurred outside of the core team developing the product. The main researcher was an active team member (participant observer), contributing during and outside meetings. The product was promoted over several months by the host organisation’s communications team.

Case A’s product was regarded as useful for researchers to promote research-derived knowledge and useful for practitioners to improve clinical practice. Ongoing use by practitioners was demonstrated in the consistent numbers of users spending several minutes per visit. Within the first 12 months, web pages were accessed 3264 times (average 272 hits/month (217–479) and 8.17 min (range 06.55–17.25) spent/visit). This, along with the positive perspective of several stakeholders and end-users interviewed: *I really do like the content…there’s a lot of valuable information on there *(AB_S3), informed the framing of Case A’s product summary label as ‘is being used’.

#### Case B

Case B’s product aimed to enable primary care practitioners to compare local versus national prescribing habits regarding drug B, as one of three aims of a 33-month project (the product was launched 9 months later than planned). The team was led by a clinically trained researcher and consisted mainly of researchers, with relatively high levels of staff turnover. The host regional department was funded nationally, with a remit to deliver two components—‘data to knowledge’ and ‘knowledge to action’—the latter of which meant that product development was, essentially, a requirement of the grant. Although over-prescription of drug B was a national priority and ‘something’ was needed to address this, respondents reported that ‘another’ product was not necessarily regarded as the required solution: *Throwing another system at them, it will certainly not go down well with all the GPs* (B_F), nor that it would necessarily reach the intended end-user: *The ones that need it the most may be the least interested, and the least motivated to do it* (B_S4).

Formal consultation of stakeholders informed product design. Influential stakeholders with the potential to shape and champion the product were recruited, but the team found it challenging to assimilate and apply feedback from *a lot of voices* (B_R), with multiple opinions *lead[ing] to confusion* (B_R). Stakeholders felt their input was treated tokenistically, *they didn’t even listen to what I said* (B_S1, paraphrased as the interview was not audio-recorded), resulting in disengagement. Relationships were rescued by the late arrival of a project manager who understood the complex interface between healthcare research and practice, making the future of the product appear brighter. The main researcher’s role was as an observer. The product was hosted by the university with a promotional campaign, including a series of articles on national and regional platforms.

The delayed release meant that it was not possible to evaluate the product’s use for the purpose of this study. Stakeholders held mixed views regarding its potential use in practice, which was reliant on overcoming hurdles including formal recruitment, data sharing and ethical approvals, and then engagement by users with the product. These considerations led to its summary label ‘might be used’: drawing firmer conclusions was inappropriate given the timing of its launch in relation to this study.

#### Case C

Case C planned to develop a product aiming to support NHS sites to collect, process and present patient experience data, as one of four aims from a 26-month project, supported by a national funding agency. A large multi-disciplinary team of researchers was led by an academic with healthcare professional experience. The PI was motivated to develop the product in order to contribute useful learning to future services. Of the eight projects funded under a single call, four planned to develop similar products.

A clear need for a product was difficult to establish, and potential end-users comprised a diffuse group of professionals. The nature of the research and its findings were not regarded as easily amenable to a ‘plug and play approach’; a single coherent, accessible and publicly available product was not delivered: *The limitation is that we’ve certainly not got a big all singing all dancing [product] that is just tomorrow going to drop into another trust and work* (C_PI_2^nd^). Mutually respectful collaboration was carried forward from previous projects and built upon during this study, with patient and public involvement (PPI) group members and NHS stakeholders working jointly to co-design and implement bespoke tools in four NHS sites. Research team members and volunteers supported the use of the products in practice. Stakeholders had myriad needs at local sites: *as the project progressed, it really became apparent the core differences between the sites and the different needs* (C_R1), which appeared difficult to distil into a product applicable more generally. The main researcher was embedded within the research team before formal observation for this study began and then acted as a participant observer.

Case C co-designed a number of bespoke tools at each NHS site. Where products were used in practice, their summary label is ‘used locally’. A cohesive, publically available product was not developed, and at the point of analysis, one tool was available for use beyond the original research. The tool was published on a website but data regarding hits and downloads were not available.

#### Case D

Case D developed a product aiming to encourage the use of evidence, as one of four workstreams from a 24-month project, funded by a national charity. The research team was led by an academic. The product was delivered to fulfil a proposal made in the funding application. The aim of the product was quite conceptual which, along with contested definitions of end-users, made specifications difficult to define.

Stakeholders were recruited from a pool of interested academics and senior managers. Formal consultation included interviews and an end-of-study workshop. The product was altered materially in response to these consultation exercises, which stakeholders welcomed. The main researcher’s role was as an observer, and they were consulted formally as part of the stakeholder engagement. An external agency was employed to design the product. The team had hoped that the funder would host the product, but it became clear after discussion that their standard policy was not to host work from the projects that they fund, so it was hosted by the university.

Positive feedback from potential end-users focussed on the *really neat* appearance and presence of a checklist, incorporated in response to stakeholder consultation which *makes it real* (D_S2). Some team members and external stakeholders who were interviewed reported mixed views of the final product *I just can’t see how it dovetails in with the current information streams, for clinicians particularly* (AD_S1). There was a lack of certainty about who end-users might be, and whether and how they might use it in practice. Numbers (see Table 3) accessing the product were closely aligned with limited early communications activity, with drop-off over time. This gradual reduction in access, along with muted reception from stakeholders interviewed for this research, informed the decision to frame the product’s summary label as ‘appears not to be used’. Table 4 details the main themes and supporting data in greater depth.

**Table 4:** Main themes and example data

Main theme	Subtheme	Description	Example from the data
Aspects of the product			
Perceived need	Product needed	Team members and stakeholders were able to identify a need for the product being developed.	E.g. one stakeholder discussing that there was a need for a product from cases A and B and comparing the two ‘in terms of population health, the need [for intervention on this topic] is probably greater for [Case B] over [Case A]’ (AB_S2).
	Product not needed	Absence of evidence for a perceived need for the product by team members or stakeholders.	‘you’d get quite a lot of discussion about what is this topic about; they often contest the term in the first place’ (D_PI_2^nd^). ‘[type of product] – always fun, the infrastructure is very helpful, but I’m not always convinced that the data feedback loops work brilliantly. I’d love to be proved wrong [laughs]’ (B_S4).
User	Clear	Team members and stakeholders were clear about who the intended user is for the product, with consistent reporting across individuals.	Observation of cases A—primary care clinicians are clearly the intended audience. Observation of case B—primary care clinicians are clearly the intended audience, with less clarity around the role of prescribing advisors.
	Unclear	Team members and stakeholders were either unable to clearly identify the intended end-user or lack of consistency between individuals.	‘One of the challenges we’re dealing with is, who is the audience for this [product]? And I guess the more audiences we have the more challenging it is to ensure it’s relevant’ (D_PI_1^st^)
Aim	Clear	Team members and stakeholders were clear about the product aim, with consistency between individuals.	Case A: consistent description of product aims across multiple interviews and observations, e.g. clearer discharge summaries, better coding of clinical encounters, ensuring that medications are restarted.
	Unclear	Team members and stakeholders unclear about the product aim or different aims described by different team members/stakeholders.	‘it’s been difficult to have a clear kind of vision of what we were aiming for’ (B_R).
Aspects of development			
Stakeholder engagement	Embedded	Core team members had implicit knowledge of the subject and requirements of end-users, some of whom were end-users themselves.	‘I think one of the major differences and where project [B] maybe falls down a bit is that actually the project [A] working group are the end-users. Whereas the project [B] didn’t really have that much end-user engagement’ (AB_S2).
	Tokenistic	Engagement with stakeholders’ erratic and feedback not consistently assimilated into product development.	‘I think that the learning from [Case B] is that there’s been a tendency to agree on something and then for the project to disappear off and create something…and bring back a product, rather than have engagement’ (B_S4).
	Co-designed	Product co-designed with end-user groups.	Case C stakeholder engagement and co-design observed (meetings) and documented (final project report) as central to team philosophy, with each site having slightly different versions of each tool.
	Consultative	Stakeholders invited to comment on iterations of the product, with feedback incorporated in a step-wise fashion.	Observation of case D stakeholder engagement, written summaries of stakeholder input and audit trail of incorporated elements.
Academic context			
Motivates product development	Perception that product proposal increases the likelihood of grant award	Products not being developed because of a specific need for them, but rather because of a perceived need for products to be developed as part of normal expectations from applied health services research.	E.g. case D: ‘They’re not going to be interested just in academic outputs, they’re interested in [products] as well… the main thing when you’re writing a research grant is you’re thinking the whole time, what’s the most likely to satisfy the funder?’ (D_PI_1^st^). E.g. case C: four of the eight projects funded under the same call, some of which ‘overlap’ (C_PI_1^st^), planned to produce toolkits arising from their research, although toolkits were not referenced explicitly in the funding brief.
	Product proposal made with aim to mobilise knowledge	Products being proposed or developed with the express aim of mobilising knowledge	E.g. case A: whole project [product development] based around mobilising knowledge regarding syndrome A E.g. case C: ‘we wanted to produce a product, or a set of products, that would be useful in these service settings’ (C_PI_1^st^).

### Cross case analysis

#### Aspects of the product influencing use

This study identified three key aspects of products from healthcare research which may be important in enhancing their use in practice: *perceived need*, *clear aim(s)* and *clarity of intended end-users*. The first aspect is a *perceived need* for the product, reinforcing previous findings that toolkits are more likely to be used in the presence of a perceived and pre-existing *need* [18]. For example, whilst there was a *perceived need* for interventions in the areas addressed by cases A and B, case B’s product was not regarded as being necessarily the right solution to the specific problem being addressed, potentially hindering its application in practice. It was observed that having clearly defined and instrumental *aims* for the products was related to a relatively increased application of the products in practice, compared to those with less clarity. Case comparison also appeared to demonstrate a relationship between having a *clearly defined and narrow intended end-user group*, and product use. For case D, for example, contested terminology used to describe end-users made stakeholder engagement problematic, as stakeholders focussed on the terminology rather than discussing the product itself. The importance of understanding the context for research products, and ensuring that they are timely, relevant and accessible has been acknowledged previously [25]. This research adds to those elements, highlighting the importance of a *perceived need, clear aim* and *clarity of end-user*, and building upon previous research which focussed on the importance of defining the target audience [12].

These key aspects of *perceived need, aim* and *end-user* appear to be interconnected, with the presence of all three appearing to be necessary (although they may not be sufficient) for the product to have the potential to be used in practice. When all three aspects were present, teams appeared better equipped to work effectively on developing the product. Where these aspects were unclear (for example case D, with less clearly defined aims and intended users), considerable time and effort were spent either trying to define them or discussing what the product might look like.

#### Aspects of development influencing use

Participants from all four cases perceived that engagement with end-users and other key stakeholders might influence product application, in line with much of the literature on knowledge mobilisation [51–56]. Case comparison offers interesting insights into the potential influence of *stakeholder engagement* upon the use of products from healthcare research.

Despite case B’s selection of high-profile stakeholders with the potential to champion the finished product, relationships with stakeholders were jeopardised by delays and a lack of communication and strategy [57, 58]. Our study provides rich comparative data to illustrate the importance of engaging stakeholders early in the research process and maintaining communication with those who might act as potential research champions, highlighted previously in relation to engaging policymakers [25].

Of all the cases, C had the most engagement with stakeholders. That it did not develop a cohesive product for wider use beyond the original research raised a question about whether engaging such a range of stakeholders and in such depth within each local site, somehow stifled this teams’ intentions to step back and develop something more generic. This question has been raised by other scholars [59]. Despite case D consulting thoroughly with stakeholders, its product appeared not to be widely used at the point of analysis, suggesting that stakeholder engagement during product development may not be sufficient to guarantee use in practice.

Case A offers an interesting counterpoint to the argument that wide stakeholder engagement is necessary to ensure a product is used in practice. Its product was the most widely used, despite relatively low levels of engagement with stakeholders outside the core project team, suggesting that stakeholder engagement may not be necessary for success (although more engagement might have enhanced its use even further). Despite high levels of emphasis in the knowledge mobilisation literature on stakeholder engagement, we are yet to see conclusive evidence that it necessarily results in findings being taken up widely [59, 60]. The phenomenon observed here might be explained as follows. Although the development of products is, by definition, a linear approach (by virtue of the information being packaged into a resource by knowledge ‘creators’ for use in practice), this was mitigated by case A taking a systems approach to developing the product [23]. The role of the clinical academic, complemented by an *implicit understanding* not only of the subject matter but, arguably more importantly, the needs of end-users and the complex system within which they operated, with a strong network and links to high-profile organisations, enabled the development of a product which overcame the limitations that come with developing something so bound to the linear approach. Those cases taking a more linear or relational approach to product development (B and D), appeared less successful.

## Discussion

### Academic context

One of the challenges resulting from the development of products within the academic context is that they are driven by the desire of, and need for, researchers to (be seen to) mobilise knowledge from their research findings, rather than necessarily due to a clear requirement for the product articulated by a well-defined group of end-users. In all four cases studied here, this meant that the three aspects of the product influencing development (*need, aim, end-user*) were approached in a non-linear fashion, and in no particular order. The development of products outside the academic context might reasonably start with defining the end-user, establishing their needs, and aligning the aims of the product with those needs, which this research suggests might lead to enhanced application in practice.

Existing literature has identified that the upward trajectory of the development of products from healthcare research is likely to continue [7, 11–13] and this research contributes to explaining why this is the case. For case B, an explicit part of the funding requirement was to turn ‘knowledge to action’, so some kind of interactive product was regarded as a necessity. For case D in particular and case C to a lesser extent, products were proposed in the bids due to a *perception* by researchers that doing so would enhance their chance of winning the award. In contrast, the *motivation for development* of case A’s product was that its development was actively sought as the only output from that specific project, which aligns with the demonstrable need for the product and its successful application in practice.

An overarching theme identified from this research is that the academic context creates a *tension between linear and systems approaches to knowledge mobilisation*. Despite (some) academics’ understanding of knowledge mobilisation principles, including the benefits of taking a third-generation, systems approach, this study highlights that the academic context forces the development of products which are more aligned with a first generation, or linear model [23]. Where a systems approach to product development was taken (case A, a relative deviant in this study), this tension was mitigated. This research provides rich evidence for the observation that, within the constraints of the current academic context, practising knowledge mobilisation principles is much harder than preaching them both for researchers [57] and healthcare research funding agencies [5]. Parallels of the influence of the academic context upon knowledge mobilisation in practice may be drawn with the mismatch between institutional and policy-driven expectations of PPI, and what researchers are able to enact in practice [61].

### Implications for future product development

The development of fewer, high-quality products which fulfil the needs of end-users might act to counter the current cynicism felt by many stakeholders in regard to products from healthcare research [18]. Asking why a particular product should be developed, whether there is a need for it, what is expected of it, who its intended users are, how it should be planned and resourced, who should be involved in that process (and how), how to encourage its use and how to sustain it, might lead end-products closer to fulfilling their presumed aim of mobilising research in ways that best support practice. When considering these questions, it should be possible to conclude that a product is not needed, and to stop the development process where appropriate, thereby avoiding the development and launch of products that are not needed and/or will not achieve their aims.

Teams developing such products should pay attention to understanding end-users and their needs. Where such understanding is lacking within the research team, the importance of meaningful engagement with stakeholders rises. Both researchers and funders might benefit from a heightened awareness that stakeholder engagement requires careful planning and resourcing (in time, effort, and money) and that assigning responsibility for nurturing relationships with stakeholders and collating and assimilating their perspectives are important to optimise their potential to contribute to and champion research products now and in the future. Funders might wish to assess the proportion of grants awarded which propose the development of products, alongside costs committed to such work. They might then try to determine the impact of products which are developed, and the proportion of projects absorbing funds without developing them, in order to understand whether the (public) money they commit to these endeavours is well spent. An alternative model is one where either external agencies or research groups with the specific skills required to develop research products are employed at the project end, once it is clear which findings might generate the greatest impact.

The urge to comply with requests from colleagues to develop a ‘how to’ guide to product development was resisted initially; if nothing else this research has shown that a ‘toolkit for product development’ is unlikely to solve many of the issues it has highlighted. However, the potential for such a resource to guide the development of research products is acknowledged, with three important caveats. Firstly, a ‘how to’ guide alone oversimplifies the conditions necessary to secure ‘success’ of a product. Secondly, paying attention to relationships and taking a systems approach to product development is likely to improve its potential application. Thirdly, following these recommendations is unlikely to ameliorate the institutional constraints of project-based funding and university reward systems, both of which currently appear to create a challenging context for product development. Table 5 presents some practical questions which were developed following this research. These questions might be asked before and during product development. They are yet to be validated or evaluated; their testing and refinement might offer a pragmatic contribution towards product development in future.

**Table 5:** Design principles for product development

The following questions offer a practical contribution to anyone considering the proposal or development of products from healthcare research and are framed around the Design Council’s Double Diamond.^a^ Please note that these questions have not been validated.	
**Discover: understand, not assume**	
• Is there a need for a product on this topic?	
• If so, what kind of product would be useful for end-users?	
• What are alternative, or additional ways, of mobilising knowledge from this research?	
• Are there existing resources that already cover this topic which you might build upon, rather than developing a new one?	
**Define: define the challenge**	
• Who are the end-user(s) for your product?	
• What message is the product trying to convey?	
• Is the research from which it is derived suitable to be packaged into a product?	
• What do you want people to do once they have accessed the product?	
• Are there other ways in which you can support them in doing this?	
• Will end-users require support in addition to the product to put the findings into practice?	
**Develop: answer a clearly defined problem**	
• Have you got a clear project plan for developing your product?	
• Who has overall responsibility for delivering this plan and finalising the product?	
• Does this person have the skills required to foster relationships with stakeholders, or is additional help needed from team members or elsewhere?	
• Are your plans for engaging with stakeholders and delivering the product realistic?	
• Are you proposing stakeholder engagement because it has to be done, or because you think it will be valuable and lead to a more effective product?	
• What kind of stakeholder engagement will you carry out?	
• How will you identify and engage stakeholders to collaborate on product development?	
• Have you set boundaries for what the stakeholder engagement aims to do, and what is out of scope?	
• Have you planned time and resources to accommodate stakeholder engagement?	
• Have you planned how you will assimilate and apply input from stakeholders and feedback the influence they have had on the product?	
• Have you considered collaborating with national, networked organisations, in the development and dissemination of the product?	
• How might your funding agency and higher education institution support in the development and dissemination of the product?	
**Deliver: testing**	
• Have you planned any end-user testing for your product?	
• Is this realistic?	
• Are you proposing end-user testing because it has to be done, or because you think it will be valuable and lead to a more effective product?	
• Will you be in a position to make changes to the product after soliciting this end-user feedback?	
**Legacy: dissemination, evaluation and sustainability**	
• Where will the product be hosted?	
• How will you publicise the product?	
• How will you keep it up to date beyond the end of the project’s funding?	
• Is further funding required to help to keep it up to date?	
• How will you evaluate whether anyone is accessing it?	
• How will you evaluate whether anyone is putting the ideas you present into practice, after having accessed the product?	
• How will you consolidate the learning from your experience of developing this product to inform future product development for yourself and other colleagues?	

^a^The Design Diamond, The Design Council, 2019, https://www.designcouncil.org.uk/news-opinion/double-diamond-15-years [Accessed 10 Nov 2021]

### Strengths and limitations

This research includes multi-stakeholder perspectives (researchers, funders, directors, product users) on product development, adding to the few existing studies using a comparative case study approach [38, 39] to explore knowledge mobilisation from healthcare research. Its prospective and longitudinal nature documents significant proportions of the research cycle. Member checking and the triangulation of data from multiple sources bolstered credibility [49].

The framing of each product studied with a ‘summary label’ regarding its use aided case comparison and enabled the use of theory. These labels were not regarded as definitive and were assigned under the understanding that their status may change over time.

That all cases were located in the same institution affects the potential transferability of the findings and is a limitation. The study included stakeholders from a wider network than the main institution, and we therefore believe that the findings are likely to be broadly applicable to products developed in similar institutions (i.e. Russell group universities within the UK, and potentially beyond) because contextual factors (e.g. funding and incentive structures; the relationship between healthcare research and practice), are fairly ubiquitous internationally. Studying a broader range of cases across a range of institutions might generate even more transferable findings. Studying similar cases using a more ethnographic approach and for longer might provide further insights into the effect of work aiming to evaluate and sustain products from healthcare research.

Relationships arising between all four authors and study participants were a potential limitation. Performing reflexivity [62, 63] was an important part of the research process, in particular for the main researcher, who acted as (participant) observer in all four cases, with the potential for relationships to influence the data and its analysis [38, 46]. Care was taken to be transparent when perceived conflicts arose. Reflexivity was practised through written reflexive notes and peer-debriefing sessions. The constructivist approach also mitigated this concern, in which the roles of all actors in generating data and conducting analysis are acknowledged.

## Conclusions

This study aimed to understand why and how products from healthcare research are developed and whether aspects of the products or their development might influence their potential application. The study identified three main themes in regard to the development of products from healthcare research. First, *aspects of the product* which might influence their use in practice, including a perceived need for the product, a clear aim and clear end-user. Second, *aspects of development* influence potential application, including stakeholder engagement. Third is the influence of the *academic context* on product development. The academic context forces the ongoing development of products from healthcare research and in doing so adheres to a linear view of knowledge mobilisation, which appears to be in tension with more relational and systems approaches to knowledge mobilisation. Only where a systems approach to product development itself is employed, do products developed to mobilise knowledge from healthcare research appear to overcome this tension.

By presenting findings based on rich qualitative data generated from a longitudinal, prospective, comparative case study of the development of products from healthcare research, including a wide range of research participants, and using existing theory to illuminate those findings, this study contributes to and expands our understanding of the development of products from healthcare research. The study is important because whilst the impact agenda is pursued and the incentive structures within higher education persist in their current state, the development of such products (currently of variable relevance to their intended end-users) is likely to continue.

### Supplementary Information

**Additional file 1.** COREQ (COnsolidated criteria for REporting Qualitative research) Checklist.

## Data Availability

The datasets generated and analysed during the current study are not publicly available due to restrictions of ethical approvals obtained for this study

## Appendix 1

### Example topic guide for first PI interview

**Table:**

**Process**	
• Talk me through the planned process of product development	
• How will you disseminate it?	
• How will you sustain it?	
• Product aim	
• Why have you chosen to develop this product?	
• What do you hope the product will do?	
• What problem are you trying to address?	
• What practice are you trying to improve?	
• What is the need for this product?	
• What would be different if this knowledge were translated successfully?	
**Stakeholders**	
• Who do you think the stakeholders are?	
• How have you identified them?	
• How do you view the role of stakeholders, and what do you expect from them?	
(a) In the project overall?	
(b) In developing the product?	
(c) How do you plan to engage with them?/What will their role be?	
• Is there a different approach for different types of stakeholders?	
• At what point did/will they become involved in the project?	
• At what point did they become involved in the design of the product?	
• What kind of interaction do you have with them?	
• Does working with stakeholders create any difficulties for either side?	
• What do you think they expect from you?	
• What are the benefits to them of being involved in the project?	
• What do you think co-design/participatory research means?	
**Impact**	
• What do you hope people will do as a result?	
• What is your definition of success?	
• How will you know whether they have done it?	
• Are you going to measure this?	
• How are you going to measure it?	
• Who else do you think it would be useful to discuss this with?	

## Appendix 2

**Table:** Final coding framework for exploratory study

Broad theme	Narrow theme
NHS context	Recognising NHS context
	Difficult to access NHS
	Use in practice or not…
Academic context	Constraints from or lack of
	Pressures
	No reward for
	Excuse for missing deadlines
	Team dispersal
	Delays
Funding	Legacy reduces priority
	Organisational priority influences
	Lack overall strategy
	Funder influencing outputs
	Cost
	Design costs
Motivation	Help secure funding current + future
	Raise profile
Product aim	Toolkit not endpoint
	Different views from different stakeholders
	Ill-defined
	Centre of the overall project?
	Lack user spec
	Initial ambition significantly reduced
	Necessary
Need	Unknown/not there
	Predetermined (e.g. did not arise from research)
	Assumed
	Arose from previous work
Experience	Understands the audience’s needs
Audience	Unclear
	Would not use it
Participatory	My contribution
	Feeling of being observed
Development	Iterative
	Scoping
	Good to get views even if the product is unfinished
Product development	Limited by software
	Arose from previous work
Process	Unclear, e.g. did not know the procedure for endorsement
	Clear work packages
	Learning for researchers
	Burdensome
	Bolt on
	Hard to develop if spec unclear
	What could be done differently
Feedback	No clear plans on how to get this
Sustainability	Checking
	Further phases
	No reward for doing so
	No strategy
Project team	Leadership Important
	Staff turnover
	Dynamics
	Role within other workstreams
Stakeholders	Slows things down
	Used appropriately
	End user within the development team
	Opinions important
	Engagement suboptimal (tokenistic vs resources)
	Not wide enough
	Focus groups hard to arrange
	Not clear who they are
	Do not know what to do with their input
Funding	Legacy reduces priority
	Organisational priority influences
	Lack overall strategy
	Funder influencing outputs
	Cost
	Design costs
Impact	Measuring difficult
	Google analytics only
	No strategy
	Not being measured
	Thoughts on how to
Anxiety/fear	
Dissemination	Not really considered
	Modes
Collaboration	Broker role
	Networking
	Learning from experts
	Strength influences success

## Abbreviations

F: Funder
L: Senior leader
NHS: National Health Service
PI: Principal investigator
PM: Project manager
R: Researcher
S: Stakeholder
